# Editorial Note: The impact of financial development on enterprise green innovation under low carbon pilot city

**DOI:** 10.1371/journal.pone.0346118

**Published:** 2026-04-01

**Authors:** The PLOS One Editors

The *PLOS One* Editors issue this Editorial Note for this article [1,2] because of concerns identified about the peer review process. Readers are advised to interpret the article [1,2] with caution.

The Editors have no evidence of any author involvement in the peer review concerns.
